# Response Efficacy and Self-Efficacy Mediated the Relationship between Perceived Threat and Psychic Anxiety among College Students in the Early Stage of the COVID-19 Pandemic

**DOI:** 10.3390/ijerph19052832

**Published:** 2022-02-28

**Authors:** Wenpei Zhang, Shankuo Xiong, Yelianghui Zheng, Jinnan Wu

**Affiliations:** Business Administration, School of Business, Anhui University of Technology, Ma’anshan 243032, China; zwpahut@ahut.edu.cn (W.Z.); shankuoxiong@ahut.edu.cn (S.X.); ylhzheng@ahut.edu.cn (Y.Z.)

**Keywords:** China, COVID-19 pandemic, Fear Appeals Theory, Social Learning Theory, threat severity, threat susceptibility

## Abstract

Applying Fear Appeals Theory and Social Learning Theory, this study aims to explore the impact of perceived threat on psychic anxiety among college students in the early stage of the COVID-19 pandemic and the mediating roles of response efficacy and self-efficacy. An empirical study was conducted using an online cross-sectional survey in the early stage of the COVID-19 pandemic in February 2020. A random sampling method was applied to administer questionnaires to 646 Chinese college students. The results showed that: (1) the perceived threat of the COVID-19 pandemic, including perceived susceptibility and severity, was positively correlated with psychic anxiety; (2) self-efficacy mediated the effect of both perceived susceptibility and severity on psychic anxiety, while the response efficacy only mediated the effect of perceived susceptibility on psychic anxiety; and (3) response efficacy and self-efficacy played a serial mediating role on the relationship between perceived susceptibility and psychic anxiety. This study elucidates the relationship between perceived threat and psychic anxiety from the perspective of cognitive appraisal of threat, showing the role positive efficacy appraisal played in reducing psychic anxiety, which could be induced by the perceived threat of major public health emergencies such as COVID-19 pandemic.

## 1. Introduction

In December 2019, unexplained pneumonia emerged in Wuhan, Hubei Province, China and exhibited a high level of contagion [1]. On 11 February 2020, the World Health Organization officially named this condition “Coronavirus disease 2019” (COVID-19) [2]. To this date, the cumulative number of confirmed cases of COVID-19 in China exceeded 40,000, with more than 7000 severe cases (data provided by the National Health Commission of the People’s Republic of China). The emergency, highly contagious, and severe nature of the COVID-19 pandemic puts people at risk, including frontline workers and those who may be vulnerable because of age, race, or ethnicity, and has plunged people into widespread anxiety, making the COVID-19 pandemic a new traumatic stressor [3,4,5,6,7,8]. Therefore, exploring the mechanism of psychic anxiety generated in the early stage of the COVID-19 pandemic can provide an important reference for coping with the mental health problems arising from such a major public health emergency.

According to the Fear Appeals Theory (FAT), when faced with a significant threat, individuals conduct two sequential parts of cognitive appraisal concerning the threat itself (resulting in perceived threat) and the efficacy of the recommended threat response (resulting in efficacy appraisal) [9,10,11]. These two parts work together to influence the individual’s emotions, and adverse appraisal results can induce negative emotions (e.g., anxiety and fear) [12]. In the early stage of the COVID-19 pandemic, in particular, the high level of infectiousness and severity of COVID-19 itself may have increased the perceived threat [13], which may have been an essential factor in inducing psychic anxiety. Further, the fact that there were few effective treatments and no vaccines against COVID-19 at this stage may have negatively affected the efficacy appraisal [13], which may be an important mechanism in affecting psychic anxiety. However, whether and how these two sequential parts of cognitive appraisal influence individuals’ psychic anxiety have received little attention [14,15,16,17].

Among the many groups affected by the COVID-19 pandemic, the college student population needs more attention, not only because their health is under threat but also because their ways of studying and living have been fundamentally altered, such as online learning and isolation [18]. These make college students more vulnerable to the adverse impact of COVID-19 threat information, creating more anxiety [19]; however, the mechanism of psychic anxiety generated during this period for college students has received less attention. Hence, this paper aims to narrow these gaps in the literature by exploring the impact of the cognitive appraisal of the threat (perceived threat and efficacy appraisal) of COVID-19 on psychic anxiety and its psychological mechanisms among college students in the early stage of the COVID-19 pandemic.

According to FAT, when individuals face a threat such as the COVID-19 outbreak, their perceived threat will be developed by the first appraisal of the susceptibility and severity towards this threat [9]. Perceived susceptibility is related to the probability of the threat occurring, such as COVID-19 infectiousness; and perceived severity is related to the severity of the threat, such as the damage to health caused by COVID-19 [10]. The higher the perceived susceptibility and severity, the more likely fear and anxiety towards the threat will develop [12]. The high level of infectiousness and severity of COVID-19 may cause perceptions of high susceptibility and high severity of the threat among college students, which may be essential factors in inducing psychic anxiety. According to this deduction and based on FAT, this study hypothesized that college students’ perceived threat (both of susceptibility and severity) of COVID-19 had a positive predictive effect on their psychic anxiety (H1).

According to FAT, after the primary appraisal, an efficacy appraisal will be developed by the secondary appraisal in terms of response efficacy and self-efficacy [10,20,21]. Response efficacy refers to the extent to which individuals believe that the response measures suggested will be effective in averting the threat [20,21], the response measures such as prevention and control measures against COVID-19 implemented by the government. In contrast, self-efficacy refers to the extent to which individuals believe they are capable of coping with the threat [21], the capacity of individuals who may be able to protect themselves from infection in the COVID-19 pandemic because of, for example, scientific precautions, muscular bodies, and other strengths. Previous research has found a negative relationship between efficacy appraisal (i.e., response efficacy and self-efficacy) and individuals’ negative emotions (e.g., anxiety) [22,23], suggesting that positive efficacy appraisal may play an important role in reducing psychic anxiety induced by COVID-19. Based on the sequential correlation between perceived threat and efficacy appraisal, we also consider the positive role of efficacy appraisal in mediating between perceived threat and psychic anxiety. It has been shown that positive efficacy appraisal may increase individuals’ confidence in being capable of coping with the threat [24,25,26], which may reduce concerns about their exposure to COVID-19 and thus decrease the negative emotions (e.g., anxiety) induced by the perceived threat (of susceptibility and severity). Therefore, this study hypothesized, based on FAT, that in the early stage of the COVID-19 pandemic, college students’ response efficacy and self-efficacy in coping with the COVID-19 threat mediated the relationship between perceived susceptibility and psychic anxiety (H2a and H3a) and the relationship between perceived severity and psychic anxiety (H2b and H3b), respectively.

Social Learning Theory (SLT) suggests a possible sequential relationship between response efficacy and self-efficacy [24,25,26]. When individuals take measures in response to a threat, if the measures themselves are effective (i.e., high response efficacy), the individuals can further judge whether they can reduce the negative impact of the threat based on their ability to implement the measures (i.e., appraisal of self-efficacy). In contrast, when the measures themselves are ineffective (i.e., low response efficacy), individuals do not need to consider self-efficacy further. This suggests that “the response efficacy expectancies have priority over self-efficacy appraisals/expectancies” [24,25]. Similarly, Ruiter, Abraham, and Kok’s (2001) process model suggested that response efficacy precedes self-efficacy [26]. Therefore, this study hypothesized, based on a combination of FAT and SLT, that in the early stage of the COVID-19 pandemic, college students’ response efficacy and self-efficacy in coping with the COVID-19 threat played a serial mediating role between perceived susceptibility and psychic anxiety (H4a) and between perceived severity and psychic anxiety (H4b).

**Figure 1:**
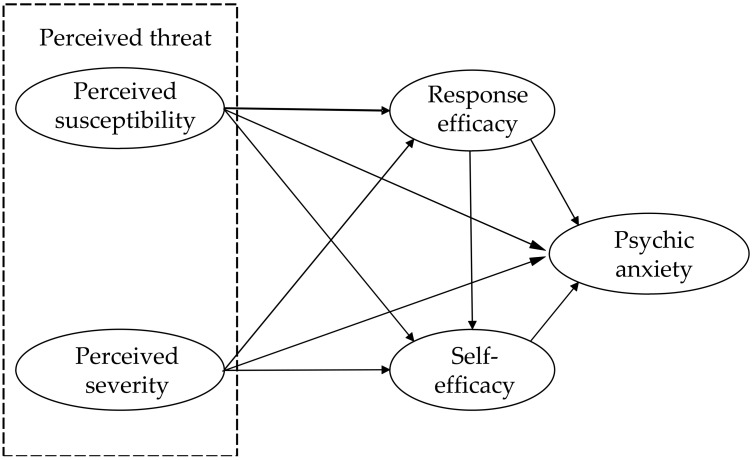
Research model.

In summary, this study integrated FAT and SLT to develop a model for understanding whether and how the cognitive appraisal of the COVID-19 threat related psychic anxiety among college students during the early stage of the COVID-19 pandemic (see Figure 1). A sample of college students was used to explore the effects of the perceived threat of COVID-19 (in terms of perceived susceptibility and perceived severity) on psychic anxiety and the mediating roles of response efficacy and self-efficacy. The findings of this study can further deepen the understanding about generating mechanisms of psychic anxiety among college students in the early stage of the COVID-19 pandemic from the perspective of the cognitive appraisal, and provide potential intervention approaches for addressing psychic anxiety among college students.

## 2. Materials and Methods

### 2.1. Participants

An online cross-sectional survey was employed for data collection. This study adopted a random sampling method and designed and implemented the survey through a reliable online survey platform (www.wjx.cn) [27] to collect data on the perceived threat, efficacy appraisal, and psychic anxiety among college students in the early stage of the COVID-19 pandemic. The participants of the survey were undergraduate students from universities in Anhui Province, and the survey was conducted from 6 to 25 February 2020. We first contacted the universities’ mental health counseling centers and student affairs departments to obtain their permission and asked them to distribute the self-assessment questionnaires online to undergraduate students in all years via a link and QR code. Written informed consent was obtained from those students before participation. The questionnaires could be accessed and filled in on a computer, mobile phone, or tablet and was limited to one response from the same IP address. A total of 678 college students completed the questionnaires; excluding samples with missing items, too-short/long response times, or too-high consistency of answers, a total of 646 valid questionnaires were finally obtained, with a valid response rate of 95.28%. According to the conventional criteria set for high statistical power, our actual sample size combined with the effect sizes as reported in this study led to high levels of statistical power [28], with the statistical power higher than 99% (f^2^ = 0.15, α = 0.05, 1-β > 0.99, using the post hoc analysis by Gpower 3.1.9.7 software) [29]. Of these, 167 (25.85%) were male and 479 (74.15%) were female. The number of participants with a health status of “good” or “very good” was 491 (76.01%), of “average” was 149 (23.06%), and of “poor” or “very poor” was 6 (0.93%). The number of participants currently living with a confirmed case count no more than 10 was 87 (13.48%), with a confirmed case count ranging from 11 to 50 was 522 (80.80%), with a confirmed case count ranging from 51 to 100 was 7 (1.08%), with a confirmed case count ranging from 101 to 500 was 25 (3.87%), and with a confirmed case count ranging from 501 to 1000 was 5 (0.77%). The participants were located in various Chinese provinces (e.g., Anhui, Jiangsu, Zhejiang, Shandong, Hunan, Hubei, Henan, Hebei, Guangdong, Gansu, Inner Mongolia, Xinjiang, etc.).

### 2.2. Measurements

To ensure the reliability of the measurements, this study applied well-established and widely used scales. The scales adopted a “translation–back–translation” approach to ensure their accuracy and were revised in the context of COVID-19. Following the approach of previous studies [15,30], three variables were controlled: sex, physical health status (PHS), and level of risk in participants’ current living area (LRPCLA).

#### 2.2.1. Perceived Susceptibility

The perceived susceptibility of COVID-19 was measured using a three-item scale, which was revised from the Perceived Susceptibility Scale [31]. All items were measured on a seven-point Likert scale, ranging from “1 = strongly disagree” to “7 = strongly agree”. These three items were “I could be infected with COVID-19”, “Someone close to me could be infected with COVID-19”, and “COVID-19 could infect many people across the country”. The Cronbach’s alpha coefficient of the scale in this sample was 0.808.

#### 2.2.2. Perceived Severity

The perceived severity of COVID-19 was measured using a three-item scale, which was revised from the Perceived Severity Scale [32]. All items were measured on a seven-point Likert scale, ranging from “1 = strongly inconsistent” to “7 = strongly consistent”. These three items were “The infection and lethality of COVID-19 threatened my life and health”, “The threat to life caused by COVID-19 was fearful”, and “The threat posed by COVID-19 made me anxious”. The Cronbach’s alpha coefficient of the scale in this sample was 0.825.

#### 2.2.3. Response Efficacy

The response efficacy was measured using a three-item scale, which was revised from the Perceived Response Efficacy Scale [33]. All items were measured on a seven-point Likert scale, ranging from “1 = strongly disagree” to “7 = strongly agree”. These three items were “All levels of government and departments of health and epidemiology can effectively control COVID-19”, “Preventive and control measures implemented by all levels of government and departments of health and pandemic prevention can prevent the spread of COVID-19”, and “Preventive and control measures implemented by all levels of government and departments of health and epidemiology can reduce the impact of COVID-19”. The Cronbach’s alpha coefficient of the scale in this sample was 0.946.

#### 2.2.4. Self-Efficacy

Self-efficacy was measured using a four-item scale, which was revised from the Perceived Self-efficacy Scale [33,34]. All items were measured on a seven-point Likert scale, ranging from “1 = strongly disagree” to “7 = strongly agree”. These four items were “I can use the right measures by myself to protect myself”, “I can use the right measures to protect myself if I can call for help”, “I can use the right measures to protect myself if I have the guidance of a government or authority”, and “I have the resources and knowledge required to take the necessary security measures”. The Cronbach’s alpha coefficient of the scale in this sample was 0.920.

#### 2.2.5. Psychic Anxiety

The Hamilton Anxiety Scale (HAMA) is one of the scales commonly used in clinical psychiatry, consisting of 14 items [35]. HAMA classifies anxiety factors into two categories: somatic and psychic. This study selected seven items related to psychic anxiety, namely from the Hamilton Psychogenic Anxiety Scale (HAMA-PSY). All items were measured on a five-point Likert scale, ranging from “1 = never” to “5 = always”, with items such as “Worries, the anticipation of the worst, fearful anticipation, irritability” and “Feelings of tension, fatigability, startle response, moved to tears easily, trembling, feelings of restlessness, inability to relax”. The Cronbach’s alpha coefficient of the scale in this sample was 0.902.

### 2.3. Statistical Analysis Strategy

SPSS 26.0 (International Business Machines Corporation, New York, NY, USA) and Mplus 7.0 (Muthén & Muthén, Los Angeles, CA, USA) were used to analyze the data. Using SPSS 26.0, means and standard deviations were calculated for the perceived susceptibility, perceived severity, response efficacy, self-efficacy, psychic anxiety, sex, PHS, and LRPCLA. Common method bias was detected by Harman’s single-factor test, correlation analyses were conducted using Pearson correlation analysis, and main effects tests were conducted by hierarchical regression. Using Mplus 7.0, a confirmatory factor analysis was performed to test common method bias and structural models further. Finally, the bias-corrected nonparametric percentile Bootstrap method was used to test for mediating effects and estimate confidence intervals.

## 3. Results

### 3.1. Common Method Bias

Since we collected data using questionnaires at a similar time from the same source, the responses needed to be tested for common method bias. Thus, an exploratory factor analysis was conducted to run Harman’s single-factor test. The results showed a cumulative variance contribution of 80.69%, with the first factor accounting for 34.54% of the variance, which was lower than the threshold of 40% [36], indicating a low probability of common method bias in the dataset. Further, we used a confirmatory factor analysis to confirm the results of Harman’s single-factor test [37]. The results showed that the fitting result of the five-factor model (*χ*^2^/*df* = 2.700, CFI = 0.974, TLI = 0.969, SRMR = 0.055, RMSEA = 0.051) was considerably better than that of the single-factor model (*χ*^2^/*df* = 37.338, CFI = 0.417, TLI = 0.336, SRMR = 0.174, RMSEA = 0.237), with Δ*χ*^2^ (Δ*df*) = 5811.542 (10), *p* < 0.001. Together, the two tests suggest that common method bias may not be a major concern in our dataset.

### 3.2. Primary Analysis

The mean values, standardized deviance, and correlations are reported in Table 1. Perceived susceptibility was negatively related to response efficacy (*r* = −0.23, *p* < 0.001) and self-efficacy (*r* = −0.39, *p* < 0.001), respectively. Perceived severity was negatively related to response efficacy (*r* = −0.13, *p* < 0.01) and self-efficacy (*r* = −0.28, *p* < 0.001), respectively. Response efficacy was positively related to self-efficacy (r = 0.46, *p* < 0.001). Response efficacy (*r* = −0.32, *p* < 0.001) and self-efficacy (*r* = −0.36, *p* < 0.001) were negatively related to psychic anxiety. These results provide preliminary evidence for our hypothesized relationships among the five constructs.

### 3.3. Main Effect Testing

Based on our hypotheses, the effects of perceived susceptibility and perceived severity on psychic anxiety were first tested. By using a hierarchical regression analysis, with controls for sex, PHS, and the LRPCLA, the relationship between perceived susceptibility/perceived severity and psychic anxiety was analyzed. The results, reported in Table 2, showed that both perceived susceptibility (*β* = 0.08, *p* < 0.05) and perceived severity (*β* = 0.18, *p* < 0.001) had a significant positive relationship with psychic anxiety, and thus, H1 was supported.

### 3.4. Structural Model Testing

After controlling the effects of sex, PHS, and the LRPCLA, we used Mplus 7.0 to test our hypothesized model. The results of the confirmatory factor analysis suggested that our hypothesized model fits well to the dataset (*χ*^2^/*df* = 2.731, CFI = 0.965, TLI = 0.959, SRMR = 0.081, RMSEA = 0.052). As we expected, perceived susceptibility was negatively related to both response efficacy (*β* = −0.156, *p* < 0.001) and self-efficacy (*β* = −0.148, *p* < 0.01). However, perceived severity was negatively related to self-efficacy (*β* = −0.127, *p* < 0.01), without being significantly related to response efficacy (*β* = −0.053, *p* > 0.05). The results further indicated that response efficacy was significantly positively related to self-efficacy (*β* = 0.407, *p* < 0.001) and that response efficacy (*β* = −0.194, *p* < 0.001) and self-efficacy (*β* = −0.213, *p* < 0.001) negatively correlated with psychic anxiety. These findings offered preliminary evidence for our mediating effects hypotheses.

### 3.5. Mediating Effects Testing

The study further examined the mediating roles of response efficacy and self-efficacy in the relationship between perceived threat susceptibility and severity and psychic anxiety using Mplus 7.0. The bias-corrected nonparametric percentile Bootstrap method was used to test for the mediating effects and estimate the confidence intervals with 2000 iterations, and the confidence interval (CI) set at 95%. Table 3 reports the results of the mediating effects. First, the mediating effects of self-efficacy (0.032; 95% CI (0.007 to 0.056)) and response efficacy (0.030; 95% CI (0.007 to 0.054)) on the relationship between perceived susceptibility and psychic anxiety was significant, both of which, not including 0, thus supported H2a and H3a. Second, the mediating effect of self-efficacy on the relationship between perceived severity and psychic anxiety was significant (0.027; 95% CI (0.002 to 0.052)), which did not include 0, thus supporting H2b; however, the mediating effect of response efficacy on the relationship between perceived severity and psychic anxiety was not significant (0.010; 95% CI (−0.009 to 0.029)), which included 0; thus, H3b was not supported. Third, the serial mediating effect of response efficacy and self-efficacy on the relationship between perceived susceptibility and psychic anxiety was significant (0.014; 95% CI (0.002 to 0.025)), which did not include 0, thus supporting H4a; the serial mediating effect of response efficacy and self-efficacy on the relationship between perceived severity and psychic anxiety was not significant (0.005; 95% CI (−0.004 to 0.013)), which included 0; thus, H4b was not supported.

## 4. Discussion

This study is the first to integrate FAT and SLT to explore the relationship between perceived threat (of perceived susceptibility and perceived severity) and psychic anxiety, and the mediating roles of response efficacy and self-efficacy among college students in the early stage of the COVID-19 pandemic. The results showed that both perceived susceptibility and perceived severity of the COVID-19 threat were significantly and positively related to psychic anxiety. Moreover, perceived susceptibility correlated with psychic anxiety through the independent mediation of response efficacy and self-efficacy, respectively, as well as through the serial mediation of response efficacy and self-efficacy. However, perceived severity correlated with psychic anxiety only through the independent mediation of self-efficacy.

The results of this study indicated that the perceived threat of COVID-19 among college students positively related to psychic anxiety; that is, college students with a higher perceived susceptibility and/or perceived severity of the threat exhibited higher levels of psychic anxiety, suggesting that perceived susceptibility and perceived severity of the threat are important factors in affecting psychic anxiety among college students during the COVID-19 pandemic [38,39]. The present study provides a comparison of evidence from China with similar previous studies, suggesting that there are no national cultural differences in the influence effect of the perceived threat of COVID-19 on psychic anxiety. However, unlike previous studies that considered the perceived threat of COVID-19 as a holistic construct, this study further refined it into two dimensions: perceived susceptibility and perceived severity, and found that both of them were important contributors to psychic anxiety among college students. This implies that the level of psychic anxiety is influenced by a combination of the individual’s perceived probability of infection with COVID-19 and the severity of the consequences of that infection.

This study also found that college students’ response efficacy and self-efficacy in coping with COVID-19 threats independently mediated the relationship between perceived susceptibility and perceived severity and psychic anxiety, respectively, but the specific pathways of these mediating effects were different. Perceived susceptibility indirectly correlated with psychic anxiety through response efficacy and self-efficacy simultaneously. In other words, the higher the perceived probability of COVID-19 infection among college students, the more likely they feel they are to be infected and, accordingly, the more difficult they think it is for either the government or themselves to cope with the COVID-19 threat, thus exhibiting their higher levels of psychic anxiety. This finding is consistent with previous studies on the mechanisms of threat perception [22,23,24,38,39].

Perceived severity could only indirectly correlated with psychic anxiety through self-efficacy, while response efficacy did not play a mediating role. That is, the higher the perceived severity of the COVID-19 threat, the lower the perceived capability of coping with the threat, and accordingly, the higher the level of psychic anxiety, which is consistent with FAT [22,23,24]. However, perceived severity of the threat was not accompanied by a reduced response efficacy, which may be explained by the effective measures taken by the Chinese government during the COVID-19 pandemic, such as the timely disclosure of detailed information about COVID-19, the refutation of rumors, and the provision of scientific advice and psychological support [40,41]. In addition, trust in the government among highly educated groups may also be important in the face of COVID-19 pandemic shocks [42].

In contrast to previous studies based on FAT [9], in which response efficacy and self-efficacy were considered as two independent mediating variables, this study creatively incorporated SLT and considered the interaction between response efficacy and self-efficacy. The results verified that response efficacy could positively relate to self-efficacy; that is, response efficacy could not only directly correlate with psychic anxiety but also correlated with reduced psychic anxiety by being linked to increased self-efficacy. Further, the serial mediating effect test results suggest that the relationship between perceived susceptibility to COVID-19 and psychic anxiety was sequentially mediated by response efficacy and self-efficacy. This finding confirmed SLT’s view that response efficacy in the face of an external stressful or threatening event contributes to an individual’s confidence in coping with the stress or threat [24,25,26]. That is, college students’ positive appraisal of the effectiveness of prevention measures against the pandemic may have an active effect on their appraisal of their own ability to cope with the pandemic and, ultimately, reduce their anxiety symptoms.

Addressing mental health in public health emergencies is vital. The results of this study recommend that, in practical interventions, authorities (e.g., governments and universities) should first focus on improving efficacy appraisal by providing psychological support to gain the trust of college students so that they believe in and comply with scientific prevention and control measures [8,41]. By inviting psychiatrists to deliver lectures, authorities can reasonably and effectively enhance the public information of COVID-19-related knowledge and scientific prevention and control measures [43]. In this way, college students can be guided to develop more positive appraisals of response efficacy and self-efficacy, thus reducing their psychic anxiety when facing major public health emergencies such as COVID-19 pandemic.

### 4.1. Strength of the Study

The initial period of a major public health emergency is the most likely period to cause widespread anxiety and panic. As the first group in the world to face the impact of the COVID-19 pandemic [44], the emotional reactions of the Chinese population in the early stage of the COVID-19 pandemic are representative; the Chinese sample during this period in this study can truly reflect the mechanism of individuals’ psychic anxiety during this particular period and, thus, has high validity. Furthermore, the results on the important role of efficacy appraisal in reducing COVID-19-induced psychic anxiety can provide effective intervention ideas for reducing psychic anxiety during similar major public health emergencies that may occur in the future.

### 4.2. Limitations of the Study

Several limitations of this study must be acknowledged. First, the study was conducted based on cross-sectional data, making it difficult to confirm the causal relationships between variables. Therefore, it is recommended that future studies consider using longitudinal research methods to verify the causal relationships. Second, the study used self-reported data, which may be affected by a social response bias; therefore, future studies may consider incorporating more objective data collection methods. Third, many confounding factors are associated with the study variables, such as personality and attitudes; however, this study only controlled for sex, PHS, and LRPCLA. In future studies, other potential influencing factors could be considered.

## 5. Conclusions

Studying people’s reactions to the threat of an unknown outbreak at the beginning of the COVID-19 pandemic can not only help us to better understand the psychic anxiety generated by the pandemic but also provide effective suggestions for coping with other similar major public health emergencies. Based on FAT and SLT, the analysis of the questionnaires data revealed that the perceived susceptibility to the COVID-19 threat positively related to psychic anxiety not only independently through response efficacy and self-efficacy but also the impact of response efficacy on self-efficacy; however, the perceived severity of the COVID-19 threat positively related to psychic anxiety only through self-efficacy. These findings reflect the important role of the perceived threat of COVID-19 in college students’ psychic anxiety and suggest a positive role of efficacy appraisal in reducing psychic anxiety, which can guide future interventions.

## Figures and Tables

**Table 1:** Mean, standard deviation, and correlations.

Variables	Mean	SD	1	2	3	4	5	6	7	8
1. Sex	N/A	N/A	1							
2. PHS	4.06	0.76	−0.05	1						
3. LRPCLA	1.98	0.61	0.06	−0.14 ***	1					
4. Perceived susceptibility	3.52	1.07	0.13 **	−0.25 ***	0.11 **	1				
5. Perceived severity	4.96	1.23	0.08 *	−0.17 ***	0.02	0.33 ***	1			
6. Response efficacy	5.90	0.89	0.06	0.24 ***	−0.02	−0.23 ***	−0.13 **	1		
7. Self-efficacy	5.60	0.88	−0.02	0.37 ***	−0.04	−0.39 ***	−0.28***	0.46 ***	1	
8. Psychic anxiety	2.07	0.69	−0.02	−0.30 ***	0.04	0.20 ***	0.25 ***	−0.32 ***	−0.36 ***	1

Notes: * *p* < 0.05, ** *p* < 0.01, and *** *p* < 0.001. SD, standard deviation; PHS, physical health status; LRPCLA, level of risk in participants’ current living area; N/A, not applicable.

**Table 2:** Results of the hierarchical regression analysis (*n* = 646).

Variables		Sex	PHS	LRPCLA	Perceived Susceptibility	Perceived Severity	Δ*F*	*R* ^2^	Δ*R*^2^
Psychic anxiety	Model 1	−0.030	−0.299 ***	0.001			21.006 ***	0.089	0.089
	Model 2	−0.053	−0.249 ***	−0.002	0.083 *	0.181 ***	17.097 ***	0.136	0.046

Notes: * *p* < 0.05 and *** *p* < 0.001. Δ*F*, *F*-test value increment; *R*^2^, *R*-square, coefficient of determination; Δ*R*^2^, *R*-square increment; PHS, physical health status; LRPCLA, level of risk in participants’ current living area.

**Table 3:** Results of the mediating effect.

Mediating Effect	Estimate	SE	95% CI	
			Lower	Upper
**Mediation of response efficacy**				
Perceived susceptibility → Response efficacy → Psychic anxiety	0.030	0.012	0.007	0.054
Perceived severity → Response efficacy → Psychic anxiety	0.010	0.010	−0.009	0.029
**Mediation of self-efficacy**				
Perceived susceptibility → Self-efficacy → Psychic anxiety	0.032	0.013	0.007	0.056
Perceived severity → Self-efficacy → Psychic anxiety	0.027	0.013	0.002	0.052
**Serial mediation of response efficacy and self-efficacy**				
Perceived susceptibility → Response efficacy → Self-efficacy → Psychic anxiety	0.014	0.006	0.002	0.025
Perceived severity → Response efficacy → Self-efficacy → Psychic anxiety	0.005	0.004	−0.004	0.013

Notes: SE, standard error; CI, confidence interval.

## Data Availability

The datasets used in this research are available upon request from the corresponding author.
